# The Prospective Association Between the Five Factor Personality Model With Health Behaviors and Health Behavior Clusters

**DOI:** 10.5964/ejop.v14i4.1450

**Published:** 2018-11-30

**Authors:** Chelsea Joyner, Ryan E. Rhodes, Paul D. Loprinzi

**Affiliations:** a Department of Health, Exercise Science and Recreation Management, Physical Activity Epidemiology Laboratory, Exercise Psychology Laboratory, The University of Mississippi, Oxford, MS, USA; b Behavioral Medicine Laboratory, School of Exercise Science, Physical and Health Education, The University of Victoria, Victoria, BC, Canada; Department of Psychology, Webster University Geneva, Geneva, Switzerland

**Keywords:** alcohol, diet, epidemiology, exercise, psychology, sleep, smoking

## Abstract

To examine the prospective association of personality with individual behavior, multibehavior and clustered health behavior profiles. A prospective study design was employed. Two hundred young adults provided baseline data and 126 (mean age: 21.6 yrs) provide complete data for a 5-month follow-up assessment (63% response rate). Personality and health behaviors (and covariates) were assessed via validated questionnaires. A multibehavior index variable was created ranging from 0-5; two separate health behavior cluster indices were created, including high (4-5 behaviors) vs. low (2 or fewer) behavior adoption and an energy balance cluster (MVPA and diet). When examining MVPA as a continuous variable, the personality trait conscientiousness was prospectively associated with MVPA and a healthy diet. Extraversion was prospectively associated with high (vs. low) behavioral clustering (OR = 1.18; 95% CI: 1.00-1.40) and conscientiousness was prospectively associated with energy balance clustering (OR = 1.09; 95% CI: 1.01-1.17). Extraversion, conscientiousness, openness to experience, and agreeableness were associated with select health-related behaviors. Further, extraversion and conscientiousness were associated with health behavior clustering.

Health-enhancing behaviors such as physical activity, smoking avoidance, non-heavy alcohol abuse, healthy eating, and adequate sleep, may help to prevent morbidity and mortality (Loprinzi, 2016a; Loprinzi, Branscum, Hanks, & Smit, 2016; Loprinzi & Mahoney, 2014; Noble et al., 2016). Further, adopting such individual health behaviors may help to delay the onset of disability and attenuate the rate of functional decline (Lee et al., 2012). Strikingly, our recent work (Loprinzi et al., 2016) demonstrates that few (<5%) adults in the United States concurrently adopts these health behaviors. Thus, there is an urgent need to promote the concurrent adoption of multiple health-enhancing behaviors, as our recent work also demonstrates that those who adopt more health behaviors tend to have better cardiovascular disease risk profiles (Loprinzi et al., 2016) and are at a reduced risk of premature mortality (Loprinzi, 2016a).

In addition to the promotion of concurrent adoption of the above-mentioned health behaviors, our work (Loprinzi, 2015a, 2016b) suggests that there may be a differential effect of unique health-behavior clusters on health status. For example, Loprinzi (2015a) demonstrated that the adoption of more health behaviors was associated with reduced odds of multimorbidity, whereas the two health behavior clusters associated with multimorbidity were physical activity and sleep as well as physical activity and nonsmoking. The differential clustering effect may also be unique to the health outcome as, recently, Loprinzi (2016b) demonstrated that the health behavior clusters of physical activity and nonsmoker as well as diet and sleep were associated with lower levels of systemic inflammation. Similarly, other work (Alzahrani, Watt, Sheiham, Aresu, & Tsakos, 2014; Duncan et al., 2014; Lee et al., 2012; Meader et al., 2016; Spengler, Mess, Mewes, Mensink, & Woll, 2012), across varying populations and health outcomes, also suggests that, in addition to the importance of concurrent adoption of more health-enhancing behaviors, health behavior clusters may have unique synergistic effects on health.

Identification of evidence-based approaches to increase the likelihood of concurrent behavioral adoption is of major public health interest. Various theoretical models, such as the Social Cognitive Theory, Transtheoretical model and Theory of Triadic Influence, provide insight on how to accomplish this (Fisher, 2008; Lippke, Nigg, & Maddock, 2012). Additionally, and as we have discussed within various behavioral domains, including physical activity (Loprinzi & Walker, 2016; Loprinzi, Wolfe, & Walker, 2015), diet (Joseph, Alonso-Alonso, Bond, Pascual-Leone, & Blackburn, 2011; Loprinzi, 2015a) and smoking (Loprinzi, Herod, et al., 2015; Loprinzi & Walker, 2016; Loprinzi, Wolfe, et al., 2015), it is plausible that changing one health behavior may help to foster changes in other health behaviors. This may occur through a variety of mechanisms, including, for example, via changes in behavior-induced cognitions (e.g., executive function; Loprinzi, 2015b) and psychological-based self-efficacy (Loprinzi, Wolfe, et al., 2015). In addition to these potential antecedents to multibehavior and behavioral clustering, personality trait characteristics plausibly play an important role in single, multibehavior, and behavioral clustering change.

To describe personality, psychologists use a widely examined theory (Big Five) that suggests there are five broad dimensions of personality. This higher order trait taxonomy characterizes each of the five personalities, including, *neuroticism* (i.e., feelings of anxiety, anger, guilt, frustration), *extraversion* (i.e., manifested in outgoing, talkative, energetic), *conscientiousness* (i.e., vigilant, careful, organized, aim for achievement), *openness to experience* (i.e., intellectual curiosity, perceptive, creative, reflective), and *agreeableness* (i.e., kind, cooperative, sympathetic, trustworthy) (Rhodes & Smith, 2006). Previous research suggests that personality is linked with a multitude of health-enhancing (e.g., physical activity, healthy eating, adequate sleep) and health-compromising behaviors (e.g., alcohol abuse, smoking) (Magee, Heaven, & Miller, 2013).

In regards to clustering of health behaviors, physical activity, like the other behaviors, is a key health behavior that has the potential to prevent numerous diseases (Brugnara et al., 2016; Hill, Gardiner, Cavalheri, Jenkins, & Healy, 2015). Therefore, it serves importance to examine specific personalities that may exhibit lower levels of physical activity. For example, and as we have discussed elsewhere in a systemic review, individuals who express high levels of neuroticism tend to be less physically active than those who express lower levels of neuroticism (Rhodes & Smith, 2006). A potential explanation for this finding is that individuals with higher levels of neuroticism tend to experience high levels of anxiety and this may pose as a potential barrier to physical activity participation. Furthermore, individuals who have higher levels of conscientiousness tend to be more physically active (Rhodes & Smith, 2006), which may be a result of their increased awareness of the importance of living an active lifestyle. The extant literature suggests there is no present evidence to associate agreeableness with physical activity (Rhodes & Smith, 2006). With regard to openness to experience, the majority of research we addressed in our review paper demonstrates a null association with physical activity (Rhodes & Smith, 2006).

Personality has also been associated with other health behaviors such as smoking, dietary behavior, alcohol use, and sleep (Magee et al., 2013). The personality trait neuroticism has been suggested to have a negative association with these health behaviors (Magee et al., 2013). In contrast, conscientiousness has been shown to favorably associate with each of these health behaviors, i.e., positive association with diet and sleep, and inverse association with smoking and alcohol (Magee et al., 2013). Extraversion, openness to experience, and agreeableness have inconclusive findings in the literature when examining the relationship to these specific health behaviors (Magee et al., 2013). Collectively, these findings suggest that those with higher levels of neuroticism are more likely to engage in health compromising behaviors such as smoking, poor diet, not meeting sleep recommendations, and a poor diet. In contrast, individuals with higher levels of conscientiousness may be more likely to engage in health enhancing behaviors such as meeting sleep recommendations, consuming a healthy diet, and participating in regular physical activity.

Research studies have examined the associations between personality and these individual health behaviors (Booth-Kewley & Vickers, 1994). Indeed, we previously demonstrated that the personality trait consciousness was positively associated with physical activity behavior (Joyner & Loprinzi, 2018). Less research has evaluated the association between personality and multibehavior as well as the clustering of health behaviors (Raynor & Levine, 2009). Thus, to extend our previous findings that just looked at a single outcome behavior (Joyner & Loprinzi, 2018), herein we utilize data from our previous work to explore the effects of personality on multibehaviors and behavioral clustering. Evaluating the concurrent adoption of multiple behaviors (multibehavior) and behavioral clustering is important, because as noted above, these behavioral patterns may have a unique influence on health, when compared to single behaviors in isolation. As a result, the purpose of this study was to overcome these gaps in the literature. Specifically, the aim of this study was to examine the prospective association of personality with individual, multibehavior and clustering of behavior engagement and behavior change. Based on findings in the literature, we hypothesize that the personality trait conscientiousness will be positively associated with health-enhancing behaviors (and clusters) and neuroticism, in particular, will be to be inversely associated with health-compromising behaviors. These findings may help to identify which personality traits may be susceptible to an overall unhealthy profile.

## Methods

### Design and Participants

The study design was a prospective study. Details on the study design can also be found in our previous work (Joyner & Loprinzi, 2018), which evaluated the association between personality and physical activity and whether executive function moderated this relationship. In the present paper, we extend these previous findings by evaluating a multitude of additional collected variables, including other behaviors (e.g., smoking, diet, sleep, alcohol). This allows for the present paper to specifically evaluate the extent to which personality may be associated with multibehavior and behavioral clustering, which is the unique contribution of the present study.

As described elsewhere (Joyner & Loprinzi, 2018), recruitment of individuals included 200 undergraduate and graduate students from a university in the South of the United States for baseline assessments. Participants were recruited via a convenience-based sampling approach. When participants arrived at the laboratory, they were asked to complete an informed consent document. Then, participants completed surveys assessing personality and the health behaviors. All baselines parameters were assessed via paper-and-pencil surveys. Baseline assessments occurred between June 2016 and October 2016. After approximately five months from the participant’s baseline visit, all participants were reassessed. To minimize potential common method bias among the evaluated parameters (Podsakoff, MacKenzie, Lee, & Podsakoff, 2003; Wingate, Sng, & Loprinzi, 2018), baseline assessments (with the exception of measured body mass index) occurred in our laboratory via a paper-and-pencil survey, whereas the follow-up participants completed the survey via an on-line portal (Qualtrics) that was sent to them via e-mail.

Among the 200 participants who were recruited, all 200 participants provided complete baseline data on the study variables (no missing values). Among these 200 participants, 126 provided complete data for the follow-up assessment (63% response rate), with these 126 participants constituting our analytic sample. When comparing (baseline estimates) the analyzed sample (*N* = 126) to the sample lost to follow-up (*N* = 74), there were no differences in gender (*p* = .61), age (*p* = .72), ethnicity (*p* = .62), education (*p* = .07), perceived health status (*p* = .50), neuroticism (*p* = .64), extraversion (*p* = .71), openness (*p* = .95), agreeableness (*p* = .59), consciousness (*p* = .75), hours of sleep per night (*p* = .86), or dietary score (*p* = .62). However, those lost to follow-up were less likely to smoke at baseline (79.1% vs. 93.6%, *p* = .002), consumed more alcoholic drinks/month (6.8 vs. 4.1; *p* = .007) and were less active (287.5 min/week of MVPA vs. 428 min/week of MVPA; *p* = .003).

Additionally, a random 10% sample from the baseline 200 participants was asked to complete a one-week test-retest reliability measure from baseline. The one-week test retest consisted of completing all of the health behavior and personality assessments for test-retest reliability purposes. The participants randomly selected for the one-week test-retest wore a pedometer for a week in an effort to assess the possible convergent validity of the self-reported physical activity assessment.

### Measurement of Personality

In order to assess personality, the Neuroticism-Extraversion-Openness Five Factor Inventory (NEO-FFI) questionnaire was used. The NEO is a five factor inventory scale. The inventory consists of five 12-item scales (60 items total) that measure each domain of five factors (Neuroticism, Extroversion, Openness, Agreeableness, Conscientiousness). There are 60 statements that describe people in a general way. A sample item from the NEO questionnaire that assesses Conscientiousness is “*I keep my belongings clean and neat*;” A sample item assessing Extroversion is “*I like to have a lot of people around me*;” A sample item assessing Openness is “*I am intrigued by the patterns I find in art and nature*;” A sample item assessing Neuroticism is “W*hen I’m under a great deal of stress, sometimes I feel like I’m going to pieces*;” and lastly, a sample item for Agreeableness is “*I would rather cooperate with others than compete with them*.” Response options range from zero to four, with zero being strongly disagree and four being strongly agree. Response option two stands for neutral. The NEO-FFI provides a quick, reliable, and accurate measure of the five domains of adult (ages 17 years and older) personality (Costa & McCrae, 1992). In our sample, internal consistency, as measured by Cronbach’s alpha, was .85 (neuroticism), .75 (extraversion), .75 (openness), .76 (agreeableness), and .84 (conscientiousness). The test-retest reliability (ICC) assessment of the 10% random sample was .92 (neuroticism), .95 (extroversion), .93 (openness), .98 (agreeableness), and .96 (conscientiousness).

### Measurement of Health Behavior

#### Physical Activity Behavior

Physical activity was assessed using the International Physical Activity Questionnaire (IPAQ). The IPAQ form asked participants about the time they spend being physically active in the last seven days. For example, a question on the form is “*How much time did you usually spend on one of those days doing vigorous physical activities in the garden or yard?*” Participants can report their answer in hours per day or minutes. Previous research shows the IPAQ to demonstrate some evidence of being reliable and valid (Craig et al., 2003). Meeting moderate-to-vigorous physical activity (MVPA) guidelines was defined as at least 150 minutes/week. Among the 10% random sample of the present study, the correlation between IPAQ-determined MVPA and pedometer-determined steps was, *r* = 0.43 (*p* < .001). The one-week test-retest reliability (for IPAQ) of our 10% random sample was, ICC = .79.

#### Smoking Behavior

Participants were classified as smokers if they self-reported smoking cigarettes every day or some days; otherwise, classified as non-smoker. Previous research demonstrates evidence of validity for self-reported smoking assessment (Yeager & Krosnick, 2010). The one-week test-retest reliability of our 10% random sample was, ICC = .91.

#### Alcohol Consumption Behavior

Participants were asked to complete a survey assessing alcohol consumption. Participants were asked whether in the past 12 months they “*had at least 12 drinks of any type of alcoholic beverage (drink = a 12 oz beer, a 4 oz glass of wine, or an ounce of liquor)?*” Those who answered “yes” to this question were further asked, “*In the past month, on those days that you drank, on average, how many drinks did you have* (responses ranging from “*I didn’t drink in the past month” to some numeric response*)? This alcohol assessment was adopted from the NHANES alcohol assessment. Although recognizing an inverted U-shaped relationship between alcohol behavior and health (Smothers & Bertolucci, 2001), due to cell size considerations, participants were ultimately scored as a heavy alcohol drinker or not, with heavy alcohol drinking defined as >30 alcoholic drinks/month for women and >60 alcoholic drinks/month for men (Arndt, Schultz, Turvey, & Petersen, 2002; Shaw, Schultz, Sperling, & Hedden, 2015). The one-week test-retest reliability of our 10% random sample was, ICC = .76.

#### Sleep Behavior

Based on the format of the Pittsburgh Sleep Quality Index questionnaire, sleep duration was assessed by asking the participants their typical nightly sleep duration over the past 30 days. Participants were classified as meeting sleep guidelines based on sleeping 7-9 hrs/night (Centers for Disease Control and Prevention, 2011). A modest correlation (*r* = 0.47) has been observed between self-reported sleep duration and objectively-measured sleep duration (Lauderdale, Knutson, Yan, Liu, & Rathouz, 2008). The one-week test-retest reliability of our 10% random sample was, ICC = .62.

#### Dietary Behavior

Participants also completed an 8 item *Starting the Conversation* (STC) dietary questionnaire, which assesses food patterns (vs. nutrient or fat intake) and has been used as a tailored approach for dietary counseling. An example item is, “*In the past week, how many times did you eat fast food meals or snacks*?” For each of the 8 items, there are three response options, which varies based on the item. As an example, response options included “< 1 time, 1-3 times and 4+” for “How many times a week did you eat fast food meals or snacks?” For the item, “How many servings of fruits did you eat each day?” response options included, “5+, 3-4, or 2 or less.” Ultimately, the summed dietary score ranged from 8-24, with higher scores reflecting a greater dietary behavior (some items reversed coded to reflect this). Given that there is no established cut-point for the STC questionnaire, this variable was dichotomized at the sample median (i.e., 18) to reflect healthier vs. less healthy dietary behavior. The STC has demonstrated evidence of feasibility, validity and sensitivity to change (Paxton, Strycker, Toobert, Ammerman, & Glasgow, 2011). The one-week test-retest reliability of our 10% random sample was, ICC = .61.

#### Multibehavior and Behavioral Clustering

A multibehavior index variable was created ranging from 0-5 indicating the number of positive health behaviors they engaged in. For example, those meeting MVPA guidelines (≥ 150 min/week), having a healthy diet (sample median score ≥ 18), meeting sleep guidelines (7-9 hrs/night), not abusing alcohol (≤ 30 alcoholic drinks/month for women and ≤ 60 alcoholic drinks/month for men) and being a non-smoker were given a multibehavior index score of 5. The one-week test-retest reliability of our 10% random sample was, ICC = .84.

In addition to this 0-5 multibehavior index variable, we created two other primary index parameters. We evaluated the association between personality types and high vs. low behavioral clustering. Taking into account cell sizes for the behaviors, high behavioral clustering was defined as having 4-5 of the health behaviors, with low behavioral clustering defined as having 2 or fewer health behaviors. Additionally, we evaluated the association between personality types and energy balance clustering. Energy balance clustering was defined as meeting physical activity guidelines and being above the median for diet behavior. Other non-primary behavior clusters were evaluated and reported in the sensitivity analyses shown in the results section.

#### Data Analysis

All analyses were performed in Stata (v. 12). Multivariable linear and logistic regression analyses were used to assess the association between baseline personalities with each of the individual health behaviors (and multibehavior as well as behavioral clusters) assessed at the 5-month follow-up period. Further, a multivariable ordinal regression model was used to assess the association of baseline personalities with the follow-up multibehavior index variable. For each of the health behaviors, two regression models were computed. For Model 1, covariates included age, gender, race-ethnicity, education, perceived health status (excellent, very good, good, fair or poor), measured baseline body mass index (kg/m^2^), and follow-up duration (months; follow-up minus baseline). Model 2 was the same as Model 1 except in Model 2 the baseline health behavior was also included as a covariate. Additionally, for the health behaviors measured in a continuous scale (i.e., diet and MVPA), a third model was computed that included the “change” score for that respective variable in the model (e.g., MVPA_time2_ – MVPA_time1_). For both models, all 5 personality traits were included in the model. In all models, there was no evidence of multicollinearity (e.g., highest individual variance inflation factor was 1.9). Statistical significance for all models was set at an alpha level of *p* < 0.05.

In an effort to minimize regression dilution bias, i.e., measurement error in the exposure, we also estimated a corrected regression coefficient by calculating a reliability ratio, as described elsewhere (Berglund, 2012). Briefly, our calculated reliability ratio was the ICC from the test-retest reliability assessment. The corrected regression beta coefficient for each personality trait (with the health behavior as the outcome) was calculated as the observed regression coefficient divided by the reliability ratio. This approach is only applicable to simple linear regression models (Berglund, 2012); thus, this approach was applied to simple linear regression models (Table 4) for diet (summed score ranging up to 24), MVPA (min/week), alcohol drinks (per month) and sleep duration (min/night of sleep); notably, this approach was not applied to the smoking data given that it was scored as a binary variable.

## Results

Study variable characteristics are displayed in Table 1.

Participants of the sample included 81% undergraduate students and the remaining 19% were graduate students. Mean age for the participants was 21.6 years, ranging from 18-33. The mean follow-up duration was 159.6 days (approximately 5.3 months), ranging from 111-241 days (approximately 3.7-8.0 months).

**Table 1:** Characteristics of the Study Sample (N = 126)

Study Variable	*M* / Proportion	*SD*
Age, mean years	21.6	2.3
Gender, % female	61.9	
Education, % undergraduate students	81.0	
Ethnicity, percent non-Hispanic white	66.0	
BMI, mean kg/m^2^	25.9	6.9
Health Status		
% excellent	16.7	
% very good	47.6	
% good	31.0	
% fair	4.8	
MVPA at baseline, mean min/week	428.0	353.3
MVPA at follow-up, mean min/week	571.5	408.5
% meets guidelines at baseline^a^	78.5	
% meets guidelines at follow-up^a^	89.7	
% non-smoker at baseline	93.6	
% non-smoker at follow-up	91.2	
Alcohol at baseline, mean drinks/month	4.1	3.9
Alcohol at follow-up, mean drinks/month	4.3	6.7
% not a heavy drinker at baseline	84.1	
% not a heavy drinker at follow-up	84.9	
Diet score at baseline, mean healthy diet	17.9	2.3
Diet score at follow-up, mean healthy diet	17.7	2.5
% healthy diet at baseline	61.1	
% healthy diet at follow-up	52.4	
Sleep at baseline, duration hrs/day	6.7	1.1
Sleep at follow-up, duration hrs/day	7.3	1.2
% healthy sleep at baseline	46.8	
% healthy sleep at follow-up	36.5	
Overall Behavior Score at baseline, mean	3.6	1.0
Overall Behavior Score at follow-up, mean	3.6	0.9
% High behavioral clustering (4-5 behaviors) at baseline	54.7	
% High behavioral clustering (4-5 behaviors) at follow-up	55.5	
Energy balance cluster at baseline, % meeting MVPA guidelines and above-median diet score	54.0	
Energy balance cluster at follow-up, % meeting MVPA guidelines and above-median diet score	50.8	
Follow-up duration, mean days	159.6	24.4

*Note.* BMI = body mass index; MVPA = moderate/vigorous physical activity.

^a^MVPA for at least 150 minutes/week. ^b^7-9 hours of sleep/day.

With regard to Model 2 (Table 2), which controlled for the respective health behavior at baseline (along with the other covariates), the personality traits extraversion, openness to experience, and agreeableness were associated with alcohol intake, respectively (OR: 1.22, 95% CI: 1.0-1.48, *p* = .05), (OR: 0.87, 95% CI: 0.76-0.99, *p* = .04), (OR: 0.84, 95% CI: 0.72-0.99, *p* = .03). The health behaviors smoking and MVPA were not significantly associated with any of the personality traits. However, when examining MVPA as a continuous variable, the personality trait conscientiousness was associated with higher MVPA (95% CI: 4.08-29.75, *p* = 0.01). The personality trait conscientiousness was associated with a healthy diet (OR: 1.11, 95% CI: 1.01-1.21, *p* = .02).

**Table 2:** Multivariable Logistic Regression Association Between Personality Type and Individual Health Behaviors

Factor	Odds Ratio (95% CI)^†^											
	Meeting MVPA Guidelines			Non-Smoker		Non-Heavy Alcohol Drinker		Healthy Diet			Meets Sleep Guidelines	
	Model 1	Model 2	Model 3	Model 1	Model 2	Model 1	Model 2	Model 1	Model 2	Model 3	Model 1	Model 2
N	1.0 (0.92-1.09)	0.97 (0.88-1.08)	7.79 (-3.4-19.0)	1.02 (0.93-1.12)	0.98 (0.88-1.10)	0.96 (0.89-1.04)	0.96 (0.87-1.06)	1.01 (0.96-1.06)	1.01 (0.95-1.07)	0.02 (-0.02-0.08)	0.96 (0.91-1.02)	0.97 (0.92-1.03)
E	1.08 (0.94-1.25)	1.0 (0.85-1.18)	-0.03 (-16.7-16.6)	0.96 (0.83-1.10)	0.89 (0.74-1.07)	**1.19** **(1.04-1.36)**	**1.22** **(1.0-1.48)**	1.04 (0.96-1.13)	1.03 (0.94-1.12)	0.04 (-0.04-0.12)	0.94 (0.86-1.02)	0.91 (0.83-1.00)
O	1.0 (0.90-1.11	0.97 (0.86-1.10)	-6.8 (-19.5-5.8)	0.96 (0.87-1.06)	0.93 (0.83-1.05)	0.93 (0.84-1.03)	**0.87** **(0.76-0.99)**	0.97 (0.91-1.03)	0.97 (0.91-1.04)	-0.01 (-0.07-0.05)	1.04 (0.98-1.11)	1.04 (0.97-1.11)
A	0.98 (0.86-1.11)	1.03 (0.89-1.18)	5.3 (-10.3-21.1)	1.09 (0.96-1.25)	1.12 (0.96-1.30)	**0.83** **(0.73-0.95)**	**0.84** **(0.72-0.99)**	0.97 (0.90-1.04)	0.96 (0.89-1.05)	-0.04 (-0.12-0.03)	1.02 (0.94-1.10)	1.04 (0.95-1.13)
C	1.02 (0.90-1.15)	0.98 (0.85-1.13)	4.4 (-10.8-19.7)	1.04 (0.92-1.17)	1.02 (0.89-1.17)	0.99 (0.88-1.11)	0.95 (0.80-1.13)	**1.11** **(1.03-1.20)**	**1.11** **(1.01-1.21)**	**0.08** **(0.005-0.16)**	0.96 (0.89-1.03)	0.96 (0.88-1.04)

*Note.* Model 1 included the following covariates: age, gender, race-ethnicity, education, health status, BMI, follow-up duration; Model 2 was the same as Model 1, but also included the *baseline* assessment of the respective health behavior; Model 3 employed a multivariable linear regression model instead of logistic regression model. This model evaluated the association of the personality traits on the “change” score in MVPA and diet. In addition to this change score variable, covariates included age, gender, race-ethnicity, education, health status, BMI, follow-up duration.

N = Neuroticism; E = Extraversion; O = Openness to Experience; A = Agreeableness; C = Conscientiousness; MVPA = Moderate-to-vigorous physical activity.

^†^For Model 3 for MVPA and Diet, the coefficients are unstandardized beta coefficients as opposed to Odds Ratios.

Bolded cells were statistically significant (*p* ≤ .05) associations.

Table 3 reports the multibehavior and behavioral clustering results. As shown in Model 1, after adjusting for baseline multibehavior, age, gender, race-ethnicity, education, health status, body mass index and duration of follow-up, neuroticism (β = -0.02; 95% CI: -0.07-0.02), extroversion (β = -0.01; 95% CI: -0.08-0.05), openness (β = -0.02; 95% CI: -0.07-0.02), agreeableness (β = -0.01; 95% CI: -0.07-0.05) and conscientiousness (β = 0.01; 95% CI: -0.04-0.07) were not associated with multibehavior. As shown in Model 2 of Table 3, extraversion was associated with high (vs. low) behavioral clustering (OR = 1.18; 95% CI: 1.00-1.40). As shown in Model 3 of Table 3, conscientiousness was associated with energy balance clustering (OR = 1.09; 95% CI: 1.01-1.17). Although not shown in tabular format, we also evaluated other types of energy balance clusters by evaluating the association personality and the concurrent adoption of meeting MVPA and sleep guidelines, as well as being above the median dietary score, but none of the personality traits were associated with this 3-variable energy balance cluster (data not shown). Similar, none of the personality traits were associated with the 2-energy balance clusters of MVPA and sleep or sleep and diet (data not shown).

**Table 3:** Multivariable Regression Models Evaluating the Association Between Personality Types and Multibehavior and Behavioral Clustering at the 5-Month Follow-up

Factor	Model 1	Model 2	Model 3
	β (95% CI)	Odds Ratio (95% CI)	Odds Ratio (95% CI)
	Multibehavior Score at Follow-Up	High Cluster vs. Low Cluster at Follow-up	Energy Balance Cluster vs. Not at Follow-up
N	-0.02 (-0.07-0.02)	1.06 (0.95-1.20)	1.00 (0.95-1.05)
E	-0.01 (-0.08-0.05)	**1.18 (1.00-1.40)**	1.04 (0.96-1.13)
O	-0.02 (-0.07-0.02)	0.99 (0.87-1.13)	0.97 (0.91-1.03)
A	-0.01 (-0.07-0.05)	0.90 (0.78-1.05)	0.96 (0.89-1.04)
C	0.01 (-0.04-0.07)	1.10 (0.95-1.26)	**1.09 (1.01-1.17)**

*Note.* N = Neuroticism; E = Extraversion; O = Openness to Experience; A = Agreeableness; C = Conscientiousness.

3 multivariate models were computed.

The first model (ordinal regression) evaluated the association between personality types and the 5 month follow-up multibehavior index score (range = 0-5) as the outcome variable. Independent variables included the 5 personality types, baseline multibehavior index score, age, gender, race-ethnicity, education, health status, BMI and follow-up duration.

The second model (logistic regression) evaluated the association between personality types and high vs. low behavioral clustering. High behavioral clustering was defined as having 4-5 of the health behaviors at follow-up, with low behavioral clustering defined as having 2 or fewer health behaviors at follow-up. Independent variables included the 5 personality types, baseline multibehavior index score, age, gender, race-ethnicity, education, health status, BMI and follow-up duration.

The third model (logistic regression) evaluated the association between personality types and energy balance clustering. Energy balance clustering was defined as meeting physical activity guidelines and being in the top median for diet behavior at the follow-up period. Independent variables included the 5 personality types, age, gender, race-ethnicity, education, health status, BMI and follow-up duration.

Bolded cells were statistically significant (*p* ≤ .05) associations.

Table 4 displays the corrected regression beta coefficients for the individual health behaviors. Notably, the slopes, for the association between each personality trait and the health behaviors, were similar when comparing the uncorrected vs. corrected slopes.

**Table 4:** Simple Linear Regression Association Between Personality Type and Individual Health Behaviors, With Correction for Regression Dilution Bias

β (95% CI)								
Factor	MVPA		Alcoholic Drinks		Diet Score		Sleep Duration	
	Model 1	Model 2	Model 1	Model 2	Model 1	Model 2	Model 1	Model 2
N	-6.39 (-14.7,1.93)	-6.94	.08 (-.05,.22)	.08	-.01 (-.06,.04)	-.01	.82 (-.76,2.4)	.89
E	**11.69 (-.36,23.7)**	12.30	**-.21 (-.4,-.01)**	-.22	.06 (-.01,.13)	.06	-.17 (-2.4,2.1)	-.18
O	-2.12 (-12.9,8.6)	-2.27	.05 (-.12,.23)	.05	-.02 (-.09,.04)	-.02	.29 (-1.7,2.3)	.31
A	3.73 (-7.8,15.2)	3.80	**-.27 (-.45,-.08)**	-.28	.02 (-.05,.09)	.02	-.09 (-2.2,2.1)	-.09
C	**15.27 (4.3,26.2)**	15.90	-.04 (-.22,.14)	-.04	**.09 (.02,.16)**	.09	-1.13 (-3.2,.99)	-1.17

*Note*. Model 1 is the unadjusted model with just the single personality type; Model 2 was the same as Model 1, but corrected for regression dilution bias.

Only the corrected regression coefficient is displayed (not the 95% CI).

N = Neuroticism; E = Extraversion; O = Openness to Experience; A = Agreeableness; C = Conscientiousness; MVPA = Moderate-to-vigorous physical activity.

Bolded cells were statistically significant (*p* ≤ .05) associations. Statistical significance was only evaluated for Model 1.

## Discussion

Previous work demonstrates that concurrent adoption of multiple health-enhancing behaviors, as well as differing clusters/combinations of these health behaviors, may have profound effects on health. Although previous research has evaluated the association of personality on individual health behaviors, mostly via cross-sectional designs, there has been limited investigation of the prospective associations of personality on multibehavior and behavioral clustering. This was the aim of this investigation. Our main findings were that: 1) select personality traits were associated with select individual health-related behaviors, 2) extraversion was associated with high behavioral clustering (i.e., engaging in the majority of the health behaviors), and 3) conscientiousness was associated with energy balance clustering. The narrative that follows will discuss our findings in the context of the individual health behaviors, followed by the multibehavior and behavioral clustering results.

When examining MVPA as a continuous variable, conscientiousness was associated with this health-enhancing behavior. Conscientiousness may serve importance as it has been suggested to be pertinent in action control (de Bruijn, de Groot, van den Putte, & Rhodes, 2009). Various studies looking at the personality trait conscientiousness have demonstrated that people with higher levels of this specific trait have better health practices and live longer (Bogg & Roberts, 2004). In alignment with this, the present study’s findings also demonstrate that conscientiousness was associated with meeting dietary behavior guidelines. Individuals with high levels of conscientiousness are thought to be more dutiful, orderly, and self-disciplined (McCrae & Costa, 1995). It is plausible to suggest that these characteristics of dutiful, orderly, and self-disciplined may play an integral part in action control and may explain why conscientiousness was associated with more physical activity and healthier eating.

The most popular personality trait model defines neuroticism as the tendency to be in a negative emotional state, anxious, self-conscious, and vulnerable (Rhodes & Smith, 2006). The results of our study demonstrated that neuroticism was not associated with any of the individual health behaviors (or multibehavior). Perhaps this is due to the levels of self-consciousness and anxiety exhibited by neurotic individuals. Research suggests that a high degree of harm avoidance or neuroticism is associated with a large activation in the insula during a risky response (Paulus, Rogalsky, Simmons, Feinstein, & Stein, 2003). The degree of risk taking is largely related to the degree of activation in the insula. Specifically, a large activation in the insula during a risky response is associated with a lower inclination to select a risky response (Paulus et al., 2003). Therefore, it is plausible to suggest that the present study did not observe any associations with the health behaviors because individuals with high levels of neuroticism tend to avoid harmful situations. Additional unexpected findings were that, for example, extraversion was associated with a higher odds of not being a heavy alcohol drinker. Our alcohol findings should be interpreted with caution as the majority of participants were not alcohol drinkers (potential floor effect).

With extraversion being characterized by the tendency to be sociable, assertive, energetic, and seek excitement, it is understandable that this personality trait was associated with high (vs. low) behavioral clustering. Providing additional plausibility for this extraversion-behavioral clustering relationship is that previous work has demonstrated that extraversion is associated with several of the health behaviors evaluated herein (Magee et al., 2013; Rhodes & Smith, 2006). Interestingly, the personality trait conscientiousness was specifically associated with energy balance clustering. Rhodes and Smith (2006) suggests that activity represents a disposition toward a fast lifestyle, representing high energy, fast talking, and keeping busy. While this facet is mainly organized under the extraversion trait, it has also been suggested as a sub-trait of conscientiousness. Costa and McCrae (1995) suggests that conscientiousness displays organizational properties and goal achievement strategies necessary for this trait to manifest. Further, conscientiousness displays self-regulation behavioral tactics which may explain why this personality trait was associated with energy balance clustering. This is in alignment with work demonstrating an indirect link between conscientiousness and dietary behavior, mediated by reduced emotional eating, restrained eating and reduced external eating (Keller & Siegrist, 2015); this suggests that highly conscientious individuals adopt regulatory dietary restraint and practice counter-regulatory emotional or external eating. In the context of the other behavior (physical activity) of this energy balance cluster, self-regulatory components (e.g., inhibition, set-shifting, goal-setting) play an important role in influencing physical activity behavior (Buckley, Cohen, Kramer, McAuley, & Mullen, 2014). The conscientiousness personality type may indirectly influence physical activity via enhanced self-regulatory abilities among such individuals (Fleming, Heintzelman, & Bartholow, 2016).

Due to the self-report nature of the health behavior assessments, our results may be limited because of social or recall bias. With the population consisting of college aged students, a limitation of this study is the limited generalizability to other ages and populations. Further, our analyzed follow-up sample differed than the initial baseline cohort for alcohol, smoking and physical activity, suggesting that our findings in these behavioral domains may have less generalizability. Notably, however, personality is considered to be stable across cultures, and therefore, the findings of this study may relate cross-culturally (Rhodes & Smith, 2006). Notable strengths of this study include the comprehensive assessment of individual, multibehavior and behavioral clusters, employing a prospective study design, incorporating a test-retest subsample (inclusive of pedometry assessment) and correcting for regression dilution bias, which is extremely uncommon in epidemiological studies (Jurek, Maldonado, Greenland, & Church, 2006). Future research would benefit by overcoming our study limitations as well as investigating lower-order personality traits (type A personality) on changes in health behaviors (Rhodes & Pfaeffli, 2012).

In order to provide substantial interventions tailored around personality type, future studies may consider utilizing a theory called Dialectical Behavior Therapy (DBT). Dialectical behavior therapy is a skills-based form of cognitive behavioral therapy and promotes acceptance of change (Lee, Cameron, & Jenner, 2015). Typically, DBT is used to treat borderline personality disorder, but research has demonstrated that it is effective in treating a wide range of issues, such as substance abuse and eating disorders. Dialectical behavior therapy focuses on teaching four sets of skills; mindfulness, distress tolerance, interpersonal effectiveness, and emotion regulation. Learning interpersonal effectiveness may be beneficial because it teaches individuals how to ask for what they want and say no while maintaining respect and relationship with others (Lee et al., 2015). Another potential personality-matched intervention tool is grounded within the Transtheoretical Model (TTM) framework. The TTM has been used to promote health behaviors and develop effective interventions (Choi, Chung, & Park, 2013; Dishman, Sallis, & Orenstein, 1985). Applying the TTM to personality traits susceptible to health-compromising behaviors may unfold the compromising behavior over time through a sequence of stages (Choi et al., 2013). Further, this model of behavior change may motivate change by enhancing the understanding of the pros and diminishing the value of the cons of unhealthy behaviors such as alcohol use, smoking, unhealthy dietary behavior, physical inactivity, and not meeting sleep guidelines. Within the framework of TTM, personality-matched strategies may include a three-step process discussed elsewhere (Conrod, Stewart, Comeau, & Maclean, 2006; Conrod et al., 2000). Briefly, personality-matched interventions may include: 1) psychoeducation, 2) behavioral coping skills training, and 3) cognitive coping skills training. With regard to psychoeducation, intervention participants are educated about the personality variable in question and the problematic coping behaviors associated with that personality style. Then, a motivational intervention (weighing the short- and long-term positive and negative consequence of a behavior) around the use of problematic behavioral strategies for coping with that personality type may be employed. Lastly, interventions can utilize cognitive coping skills involved in identifying and challenging personality-specific cognitive distortions regarding the behavior of interest. A final note is that, most believe that personality is a stable “enduring” trait that is immutable, based on personality-based stability coefficients in the range of 0.7-0.8 (McCrae & Costa, 2003). However, this ~35-50% of unexplained variance questions this immutability of personality trait change. As stated previously, using psychoeducation, behavioral and cognitive coping strategies matched per the participant’s personality trait may be useful intervention strategies. Additionally, a “bottom-up” model of change may serve to be highly effective, which targets changing basic personality processes, which may help to eventuate changes at the trait level (Chapman, Hampson, & Clarkin, 2014).

In conclusion, personality traits were differentially associated with select health behaviors; extraversion was associated with high behavioral clustering; and conscientiousness was associated with energy balance clustering. The strength of these observed associations were relatively small, which is in alignment with other personality-behavior studies. Determining personality types may be useful in identifying at risk populations. The results from this study suggest that when evaluating individual, multiple and behavioral clusters among the college aged population, it may serve importance to consider personality assessment.
